# Outcomes of Semaglutide Use in Achieving Target Body Mass Index Before Renal Transplant in Five End-Stage Renal Disease Patients: A Case Series

**DOI:** 10.7759/cureus.71511

**Published:** 2024-10-15

**Authors:** Naeem Al-saad, Gaurang Hasmukhbhai Suhagiya, Badar Ud Din Shah, Jahanzeb Malik, Syed Muhammad Jawad Zaidi

**Affiliations:** 1 Acute Medicine, Medway National Health Service (NHS) Foundation Trust, Gillingham, GBR; 2 Medicine, Jiangsu University, Zhenjiang, CHN; 3 Nephrology, Geisinger Medical Center, Danville, USA; 4 Electrophysiology, Pakistan Air Force (PAF) Hospital, Islamabad, PAK; 5 Pediatric Emergency Medicine, Shaukat Khanum Memorial Cancer Hospital and Research Centre, Lahore, PAK

**Keywords:** end-stage renal disease (esrd), obesity, renal transplantation, semaglutide, weight loss

## Abstract

Obesity is a significant barrier to renal transplantation due to associated surgical risks and postoperative complications. This case series presents five cases of obese patients with end-stage renal disease (ESRD) who successfully achieved substantial weight loss using semaglutide, a glucagon-like peptide (GLP) type-1 receptor agonist, thereby becoming eligible for transplantation. Each patient experienced significant weight reduction, ranging from 11.7% to 14.8% of their baseline weight, with minimal side effects. Semaglutide was well-tolerated, and careful monitoring prevented complications such as fluid overload. These cases highlight the potential of semaglutide as an effective and safe adjunct for weight loss in ESRD patients, offering a viable alternative to lifestyle interventions and bariatric surgery. The findings suggest that semaglutide could broaden the pool of eligible transplant candidates and improve patient outcomes by using semaglutide as a weight loss therapy. Further research is warranted to explore its long-term effects on transplant outcomes and to develop guidelines for its use in clinical practice.

## Introduction

Obesity is a significant concern in the context of renal transplantation, with over 1.9 billion adults globally classified as overweight and more than 650 million as obese [1]. Obesity presents major challenges for transplantation due to complications associated with increased surgical depth and higher rates of wound issues [2]. Body mass index (BMI) is not a perfect predictor of long-term outcomes; however, it remains a critical factor in assessing the risk of postoperative complications. Studies have highlighted that patients with a BMI over 30 kg/m² face higher risks of mortality, graft rejection, delayed graft function, and graft loss [2-4].

Lifestyle changes have generally proven difficult, and weight loss surgery can be hard to access and carries risks. Glucagon-like peptide 1 (GLP-1) receptor agonists offer a potential alternative [4]. These drugs aid weight loss by affecting neurohormonal pathways (which involve second messenger pathways and ionic events in the autonomic nervous system), slowing gastric emptying, and increasing feelings of fullness, regardless of food intake [5]. They have shown promise in achieving sustained weight loss in diabetic and non-diabetic individuals. Although these medications are not cleared through the kidneys, they have been excluded from trials involving end-stage renal disease (ESRD) patients or those on dialysis [6]. However, registry data from various studies suggest that GLP-1 receptor agonists can be used safely in ESRD patients [7-9]. There is limited evidence of their use for weight loss before renal transplantation, with only one report documenting successful outcomes.

## Case presentation

Case 1

A 45-year-old man of Southern Punjab descent is on hemodialysis for ESRD secondary to type 2 diabetes and diabetic nephropathy, diagnosed in 2015. Comorbidities include hypertension, hyperlipidemia, and obesity. Personal history includes familial type 2 diabetes. He was on insulin, metformin, lisinopril, atorvastatin, and erythropoietin.

Despite being on dialysis, he was not listed for a transplant due to class 3 obesity. At the time of assessment, his weight was 130 kg, height 176 cm, and BMI 41.9 kg/m². His target weight, set by the transplant team, was 110 kg. Semaglutide 0.5 mg weekly was started in January 2023, increasing to 1 mg after six weeks. After eight months of treatment, he achieved a weight of 111 kg, marking a 19 kg loss, or a 14.6% reduction. His weight loss was steady at 2.4 kg per month. Semaglutide was discontinued once his target weight was reached. There were no significant adverse effects, and he remained stable on dialysis with no additional complications or hospitalizations. He was added to the transplant list in September 2023.

Case 2

A 60-year-old woman of Kashmiri background with ESRD from polycystic kidney disease was diagnosed in 2018. Comorbidities include coronary artery disease, chronic obstructive pulmonary disease (COPD), and obesity. No significant family history of metabolic disorders. The medications are the following: lisinopril, carvedilol, furosemide, and vitamin D.

She was considered unsuitable for transplantation due to class 2 obesity. Her weight at evaluation was 135 kg, height was 165 cm, and BMI was 49.6 kg/m². Her target weight was 120 kg. Starting in February 2023, she was prescribed semaglutide 0.25 mg weekly, increasing to 0.5 mg after one month. Over eight months, she lost 16 kg, achieving a weight of 119 kg, a 12% reduction. Her weight loss rate averaged 2 kg per month. There were no major side effects or complications. She achieved her target weight by October 2023 and was wait-listed for a transplant.

Case 3

A 40-year-old man of Punjabi descent with ESRD due to IgA nephropathy. He has a history of hypertension, gout, and moderate obesity. Medications include allopurinol, amlodipine, and sevelamer.

His initial weight was 142 kg, height was 182 cm, and BMI was 42.7 kg/m², with a target weight of 125 kg. Semaglutide was initiated at 0.5 mg weekly in March 2023 and increased to 1 mg after six weeks. Within eight months, he lost 21 kg, achieving a weight of 121 kg (a 14.8% reduction). The average weight loss rate was 2.6 kg per month. He tolerated the medication well, with no significant side effects. After achieving his target weight in November 2023, he was listed for transplantation.

Case 4

A 55-year-old woman of Punjabi descent with ESRD secondary to hypertension and diabetic nephropathy. She has a history of obstructive sleep apnea and hyperlipidemia. Medications include metformin, simvastatin, and lisinopril.

Her weight was 128 kg, height was 170 cm, and BMI was 44.4 kg/m². The target weight was set at 115 kg. Semaglutide was started at 0.5 mg weekly in April 2023, increasing to 1 mg after six weeks. After eight months, she achieved a weight of 113 kg, a 15% reduction from baseline. Her weight loss averaged 2.3 kg per month. She experienced minimal gastrointestinal side effects and had no adverse events related to her dialysis. By December 2023, she was successfully wait-listed for a transplant.

Case 5

A 30-year-old man of Punjabi descent with ESRD due to focal segmental glomerulosclerosis (FSGS). His comorbidities include hypertension and mild obesity. No family history of metabolic disorders. Current medications include ramipril and calcium carbonate.

At evaluation, his weight was 120 kg, height was 180 cm, and BMI was 37.0 kg/m². His target weight was 105 kg. Semaglutide was prescribed at 0.5 mg weekly starting in May 2023, with an increase to 1 mg after six weeks. Over eight months, he achieved a weight of 106 kg, representing a 14 kg loss, or 11.7% reduction from his baseline. His average weight loss rate was 1.9 kg per month. The medication was well-tolerated with no significant side effects. There were no complications from rapid weight loss. By January 2024, he was added to the transplant list.

A summary of the clinical attributes of these five cases is shown in Table 1.

**Table 1:** Summary of cases in terms of baseline profile and treatment outcome COPD: chronic obstructive pulmonary disease, eGFR: estimated glomerular filtration rate, ESRD: end-stage renal disease, FSGS: focal segmental glomerulonephritis, HbA1c: glycosylated hemoglobin, PTH: parathyroid hormone

Characteristic	Case 1	Case 2	Case 3	Case 4	Case 5
Age (years)	45	60	40	55	30
Gender	Male	Female	Male	Female	Male
Ethnicity	Southern Punjab	Kashmiri	Punjabi	Punjabi	Punjabi
Diagnosis	ESRD secondary to type 2 diabetes	ESRD from polycystic kidney disease	ESRD due to IgA nephropathy	ESRD secondary to hypertension and diabetic nephropathy	ESRD due to FSGS
Year of diagnosis	2015	2018	-	-	-
Comorbidities	Hypertension, hyperlipidemia, obesity	Coronary artery disease, COPD, obesity	Hypertension, gout, obesity	Hypertension, obstructive sleep apnea, hyperlipidemia, obesity	Hypertension, mild obesity
Family history	Familial type 2 diabetes	None	None	None	None
Medications	Insulin, metformin, lisinopril, atorvastatin, erythropoietin	Lisinopril, carvedilol, furosemide, vitamin D	Allopurinol, amlodipine, sevelamer	Metformin, simvastatin, lisinopril	Ramipril, calcium carbonate
Creatinine (mg/dL)	8.5	9.2	7.8	8.1	9
eGFR (mL/min/1.73 m²)	6	5	7	6	5
HbA1c (%)	8	7.5	6.9	7.2	6.8
Calcium (mg/dL)	8.6	8.8	8.7	8.9	8.5
Phosphate (mg/dL)	5.5	5.7	5.6	5.4	5.8
PTH (pg/mL)	450	500	470	490	460
Weight (kg)	130	135	142	128	120
Height (cm)	176	165	182	170	180
BMI (kg/m²)	41.9	49.6	42.7	44.4	37
Target weight (kg)	110	120	125	115	105
Starting semaglutide	January 2023	February 2023	March 2023	April 2023	May 2023
Initial dose	0.5 mg weekly	0.25 mg weekly	0.5 mg weekly	0.5 mg weekly	0.5 mg weekly
Increased dose	1 mg after six weeks	0.5 mg after one month	1 mg after six weeks	1 mg after six weeks	1 mg after six weeks
Duration to target	Eight months	Eight months	Eight months	Eight months	Eight months
Weight loss (kg)	19	16	21	15	14
Weight loss (%)	14.60%	12%	14.80%	11.70%	11.70%
Weight loss rate (kg/month)	2.4	2	2.6	2.3	1.9
Transplant list date	September 2023	October 2023	November 2023	December 2023	January 2024
Side effects	None	Minimal gastrointestinal side effects	None	Minimal gastrointestinal side effects	None
Complications	None	None	None	None	None

All patients except case 1 underwent successful renal transplants with no complications. All patients except case 1 had successful weight loss and better glycemic control, so it was decided to continue semaglutide as a part of their treatment regimen even after the transplant. No rebound weight was noticed as semaglutide was continued in all patients except case 1.

## Discussion

The cases presented highlight the successful use of semaglutide, a GLP-1 receptor agonist, as an adjunct for weight loss in patients with ESRD who were candidates for renal transplantation but were previously ineligible due to obesity [10]. Obesity remains a significant barrier to transplantation due to the associated surgical risks and postoperative complications. The findings from these cases demonstrate that semaglutide can be an effective and well-tolerated option for achieving weight loss in this population, potentially enabling more patients to become eligible for transplant listing and ultimately improving their quality of life and long-term outcomes [3,11].

In all five cases, semaglutide therapy led to substantial weight loss, enabling the patients to reach their target weights and subsequently be listed for transplantation. The average weight loss observed across these cases was significant, with reductions ranging from 11.7% to 14.8% of baseline body weight. This is consistent with findings from clinical trials and other real-world studies where GLP-1 receptor agonists have shown efficacy in promoting weight loss in both diabetic and non-diabetic populations [5,12]. The average monthly weight loss rates of approximately 2.1 to 2.6 kg further underscore the effectiveness of semaglutide in facilitating gradual, sustained weight reduction.

Semaglutide was well-tolerated among the patients, with minimal side effects reported. The most common adverse effects associated with GLP-1 receptor agonists, such as gastrointestinal disturbances, were either absent or mild and transient. This is particularly important in the ESRD population, where gastrointestinal symptoms could potentially exacerbate existing comorbidities or interfere with dialysis [13]. The careful monitoring and adjustment of target dialysis weights to prevent fluid overload during rapid weight loss were critical in avoiding complications, and no adverse outcomes related to volume status were observed.

The use of semaglutide in these cases allowed for successful weight reduction and listing for renal transplantation. Obesity is a modifiable risk factor, and achieving a suitable BMI can significantly impact surgical outcomes and post-transplant recovery [14]. The ability to reduce weight through pharmacological means offers a viable alternative to lifestyle interventions and bariatric surgery, both of which present challenges in terms of efficacy, accessibility, and safety [11-14]. Semaglutide, therefore, represents a promising tool in the pre-transplant management of obese patients with ESRD. Dietary modifications and liposuction can play a role in weight loss; however, liposuction has an increased risk of weight gain, and dietary modifications have issues with compliance in such patients [11,13,14].

These cases suggest that incorporating semaglutide into the pre-transplant care regimen for obese patients with ESRD could potentially broaden the pool of eligible transplant candidates [5,6]. This could have significant implications for transplant programs and healthcare systems by improving patient outcomes and optimizing the use of available organs [9]. Future studies should aim to further investigate the long-term effects of semaglutide on transplant outcomes, including graft function and patient survival, as well as its impact on metabolic parameters and overall quality of life. Additionally, further research is warranted to explore the safety and efficacy of semaglutide in a larger, more diverse cohort of ESRD patients, including those with varying degrees of kidney function and different comorbidities. The development of guidelines and protocols for the use of GLP-1 receptor agonists in the renal transplant population could facilitate their broader adoption in clinical practice.

Having a small sample size and single-centered case series are some of the limitations of our case series. Multi-centered studies with good sample sizes can help in understanding the safety and efficacy of semaglutide in patients with obese patients with ESRD undergoing renal transplant. Nonetheless, our study provides baseline data for further study on the topic in obese pre-renal transplant patients.

## Conclusions

The presented cases illustrate the potential of semaglutide as an effective and safe adjunct for weight loss in obese patients with ESRD, enabling them to qualify for renal transplantation. This pharmacological approach offers a promising alternative to traditional weight loss methods, addressing a critical barrier to transplantation and improving the prospects for successful long-term outcomes in this vulnerable population. Further research is required to explore the safety and efficacy of semaglutide in a larger, more diverse cohort of ESRD patients, including those with varying degrees of kidney function and different comorbidities.
